# Integrating single-cell RNA sequencing and bulk RNA sequencing data to predict acute respiratory distress syndrome in sepsis patients

**DOI:** 10.1016/j.gendis.2024.101271

**Published:** 2024-03-19

**Authors:** Miao Wang, Xiaoer Jin, Qingbo Liao, Yufan Pu, Xiaowen Xu, Xiaoqiang Ren, Gaoqing Liu, Zhiwei Zhuang, Qi Ding

**Affiliations:** a The Affiliated Suzhou Hospital of Nanjing Medical University, Suzhou, Jiangsu 215000, China; b Gusu School, Nanjing Medical University, Suzhou, Jiangsu 215000, China; c Department of Emergency, Suzhou Municipal Hospital, Suzhou, Jiangsu 215000, China; d First Affiliated Hospital of Suzhou University, Suzhou, Jiangsu 215000, China

Sepsis affects approximately 20%–30% of patients admitted to the intensive care unit.^1^ Acute respiratory distress syndrome (ARDS) is recognized as one of the earliest and most common complications of sepsis, occurring when sepsis triggers a systemic infection and provokes an uncontrolled inflammatory response that can lead to severe lung damage.^2^ Studies have demonstrated that patients with sepsis-induced ARDS face not only a mortality risk ranging from 30% to 40%^3^ but also long-term outcomes such as cognitive impairment and memory loss.^4^ Moreover, patients with sepsis-associated ARDS have a higher mortality rate compared with those with ARDS caused by other factors and tend to have suboptimal treatment outcomes once ARDS develops.^5^ Therefore, early identification and treatment initiation are crucial to prevent ARDS in sepsis, reduce mortality, and minimize healthcare costs. In this study, we aimed to develop a predictive model for assessing the likelihood of ARDS development in sepsis patients by integrating bulk RNA sequencing and single-cell RNA sequencing data (materials & methods can be found in supplementary data).

After quality control of single-cell RNA sequencing data of seven peripheral blood mononuclear cells, we employed the “SingleR” package to identify marker genes for clustering annotation. This analysis revealed the presence of seven distinct cell clusters, namely T_cells, Monocytes, B_cells, NK_cells, Platelets, GMP, and Pre-B_cells_CD34- (Fig. 1A). Subsequently, we calculated the proportions of various cell subpopulations in sepsis and sepsis-induced ARDS and found that the proportions of T cells (sepsis *vs*. sepsis-induced ARDS: 0.37 *vs*. 0.38) and B cells (sepsis *vs*. sepsis-induced ARDS: 0.07 *vs*. 0.11) were moderately elevated in the sepsis-induced ARDS group, whereas the proportions of natural killer (NK) cells (sepsis *vs*. sepsis-induced ARDS: 0.11 *vs*. 0.08) and monocytes (sepsis *vs*. sepsis-induced ARDS: 0.43 *vs*. 0.41) were lower (Fig. 1B, C). To gain more granularity, further clustering and downscaling analyses of NK cells, monocytes, and T cells were conducted. The results revealed that patients with sepsis-induced ARDS had a significant increase in Cluster 1 NK cells (sepsis *vs*. sepsis-induced ARDS: 0.35 *vs*. 0.61) and Cluster 0 T cells (sepsis *vs*. sepsis-induced ARDS: 0.45 *vs*. 0.52) (Fig. 1D–F). Moreover, there was a significant alteration in the CD14^+^ monocyte phenotype (Cluster 0, 1, 3, and 4) and an increase in the number of CD16^+^ monocytes (Cluster 2, sepsis *vs*. sepsis-induced ARDS: 0.08 *vs*. 0.09) compared with the sepsis group. To characterize the cell subsets, 282 genes were obtained using the FindAllMarkers method.

**Figure 1:**
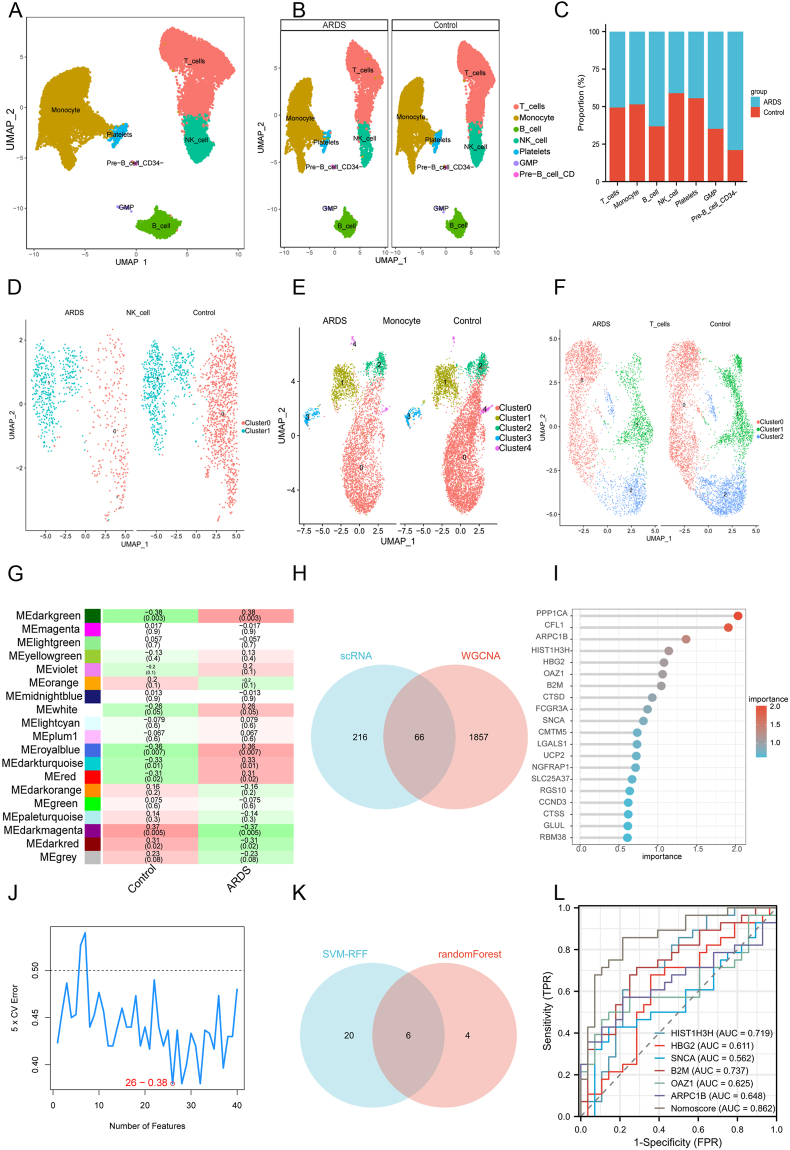
Construction of predictive model for sepsis-induced acute respiratory distress syndrome (ARDS). **(A)** Seven cell populations were annotated by singleR as T_cells (*n* = 9153), Monocytes (*n* = 10398), B_cells (*n* = 2050), NK_cells (*n* = 2388), Platelets (*n* = 396), GMP (*n* = 58), and Pre-B_cells_CD34- (*n* = 31). **(B, C)** Percentage of different cells in sepsis and sepsis-induced ARDS: T_cells (0.37 *vs*. 0.38), Monocytes (0.43 *vs*. 0.31), B_cells (0.07 *vs*. 0.11), NK_cells (0.11 *vs*. 0.08), Platelets (0.02 *vs*. 0.01), GMP (0.002 *vs*. 0.003), and Pre-B_cells_CD34- (0.0008 *vs*. 0.03). **(D)** Sepsis-induced ARDS had a significant increase in Cluster 1 NK cells (sepsis *vs*. sepsis-induced ARDS: 0.35 *vs*. 0.61). **(E)** There was a significant alteration in the CD14^+^ monocyte phenotype (Cluster 0, 1, 3, and 4) and an increase in the number of CD16^+^ monocytes (Cluster 2, sepsis *vs*. sepsis-induced ARDS: 0.08 *vs*. 0.09) compared with the sepsis group. **(F)** Sepsis-induced ARDS had a significant increase in Cluster 0 T cells (sepsis *vs*. sepsis-induced ARDS: 0.45 *vs*. 0.52). **(G)** Correlating the modules with sepsis-induced ARDS. **(H)** The intersection of single-cell RNA marker genes and genes with modular genes obtained from weighted gene co-expression network analysis is shown by Venn diagrams. **(I)** A random forest plot was utilized to analyze the relative importance of the 66 genes. **(J)** Support vector machine recursive feature elimination (SVM-RFE) algorithm identified 26 genes out of the 66 genes. **(K)** The intersection of random forest plot and SVM-RFE algorithm. **(L)** The predicted model area under the curve (AUC) was 0.838.

The occurrence of sepsis-induced ARDS was investigated using weighted gene co-expression network analysis (WGCNA) from bulk RNA sequencing data. During the analysis, four (dark green, royal blue, dark turquoise, and red) contained 1923 genes and exhibited the highest correlation with sepsis-induced ARDS scores (Fig. 1G). There were 66 candidate genes for subsequent model construction by overlapping single-cell RNA and WGCNA (Fig. 1H). Subsequently, we employed two machine learning methods, namely random forest graph analysis and support vector machine recursive feature elimination (SVM-RFE), to screen the 66 candidate genes for feature genes to be used in model construction. The random forest plot was utilized to analyze the relative importance of the 66 genes, and the top 10 genes in terms of relative importance were selected for subsequent analysis (Fig. 1I). Furthermore, the SVM-RFE algorithm identified 26 genes out of the 66 genes (Fig. 1J). Ultimately, six genes were found to overlap between the two algorithms: ARPC1B, B2M, HBG2, HIST1H3H, OAZ1, and SNCA (Fig. 1K). The area under the curve was found to be 0.838, indicating that the model's prediction outperformed that of the six signature genes alone (Fig. 1L).

We also performed functional enrichment analysis of 66 candidate genes and six sepsis-induced ARDS signature genes in single-cell RNA seq data. The findings showed higher sepsis-induced ARDS scores compared with sepsis, particularly in monocytes and NK cells (Figs. S1A–C). These findings suggest a close association between candidate genes and the function of monocytes and NK cells, highlighting the importance of these cell populations in candidate gene analysis. Additionally, we assessed the expression of six sepsis-induced ARDS signature genes across different cell clusters. The findings indicated widespread expression of SNCA, B2M, OAZ1, and ARPC1B in peripheral blood mononuclear cells, with B2M showing significantly higher expression in monocytes and NK cells, particularly in the sepsis-induced ARDS group (Figs. S1D–F). Furthermore, compared with T cells, the expression of B2M in monocytes and NK cells was significantly higher (Fig. S1E, F). Subsequently, we delved into exploring the specific functions and related mechanisms of B2M in NK cells.

Based on the mean B2M expression, the NK cells were divided into two groups. Figure S2A illustrates that the proportion of NK cells with high B2M expression significantly increased in the sepsis-induced ARDS group compared with the sepsis group. This observation suggests that NK cells exhibiting high B2M expression play a crucial role in the progression of sepsis-induced ARDS. Using FindAllMarkers and Wilcoxon tests, we identified 628 significantly differentially expressed genes between B2M high- and low-expressing NK cells. Notably, IL32, TRAC, CD53, ACTB, and S100A11 were significantly up-regulated in B2M high-expressing NK cells, whereas CD69, NFKBIA, JUN, DUSP1, and DUSP3 were significantly down-regulated (Fig. S2B). Functional analyses, including GO and KEGG, revealed significant enrichment of these differentially expressed genes in various pathways, such as ribosome structure, oxidoreduction-driven active transmembrane transporter activity, electron transfer activity, NADH dehydrogenase activity, and several disease-related pathways (Fig. S2C). Additionally, irGSEA analysis demonstrated that TGF-beta-signaling and TNFA-signaling-via-NFKB signaling were inhibited in B2M high-expressing NK cells, while reactive-oxygen-species-pathway, protein-secretion, PI3K-AKT-mTOR-signaling, and other pathways were significantly activated (Fig. S2D). Pseudo-time trajectory analysis indicated that B2M high-expressing NK cells were in a more advanced stage of differentiation (Fig. S2E, F). To further investigate the gene expression profile and potential cellular functions of NK subpopulations in pseudo time, we employed a branch expression analysis model followed by hierarchical clustering analysis. This approach allowed us to identify four distinct gene expression modules, with “cell fate 1” branch cells exhibiting high expression of IL32 and S100A11 (Fig. S2G). These genes were primarily associated with cytokine signaling in the immune system, regulation of leukocyte activation, and respiratory burst functions.

Together, we integrated single-cell RNA sequencing and bulk RNA sequencing data and applied machine learning to identify six signature genes and create a robust prediction model for sepsis-induced ARDS. Future multi-center experiments with large samples are needed to validate the predictive efficacy of these signature genes for sepsis-induced ARDS.

## Author contributions

Miao Wang, Xiaoer Jin, Qingbo Liao, and Yufan Pu: conceptualization, methodology, investigation, and writing—original draft. Xiaowen Xu: formal analysis and writing—review & editing. Xiaoqiang Ren and Gaoqing Liu: formal analysis and writing—review & editing. Zhiwei Zhuang and Qi Ding: conceptualization, supervision, and writing—review & editing.

## Conflict of interests

The authors have no competing interests to declare.

## Funding

This study was supported by the Gusu Talent Project of Suzhou Health Commission (Jiangsu, China) (No. GSWS2020065).

## Figures and Tables

**Figure S1:**
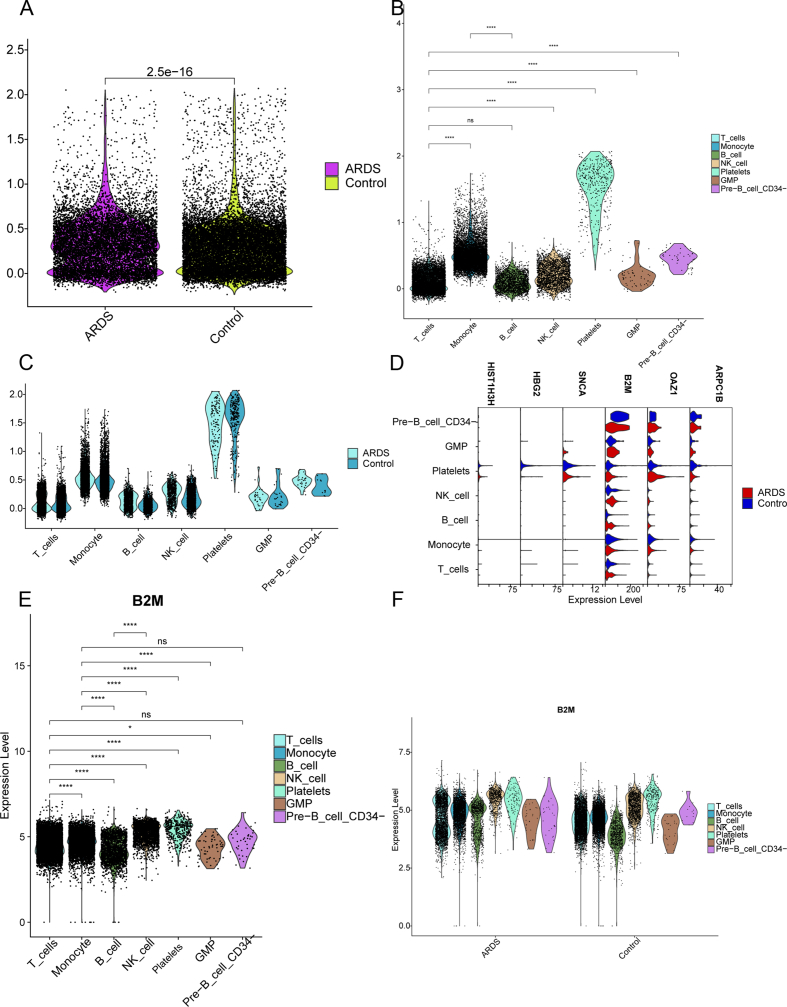
The scores of 66 candidate genes and six sepsis-induced acute respiratory distress syndrome (ARDS) signature genes in single-cell RNA sequencing data. **(A)** Higher scores in the sepsis-induced ARDS group versus the sepsis group. **(B)** Monocytes and natural killer (NK) cells exhibited higher scores than T cells. **(C)** Within monocytes and NK cells, the scores in the sepsis-induced ARDS group were significantly higher than those in the sepsis group. **(D)** Six sepsis-induced ARDS signature genes in single-cell RNA sequencing data. **(E)** The expression of B2M in monocytes and NK cells was significantly higher. **(F)** The expression of B2M in monocytes and NK cells was significantly higher in the sepsis-induced ARDS group

**Figure S2:**
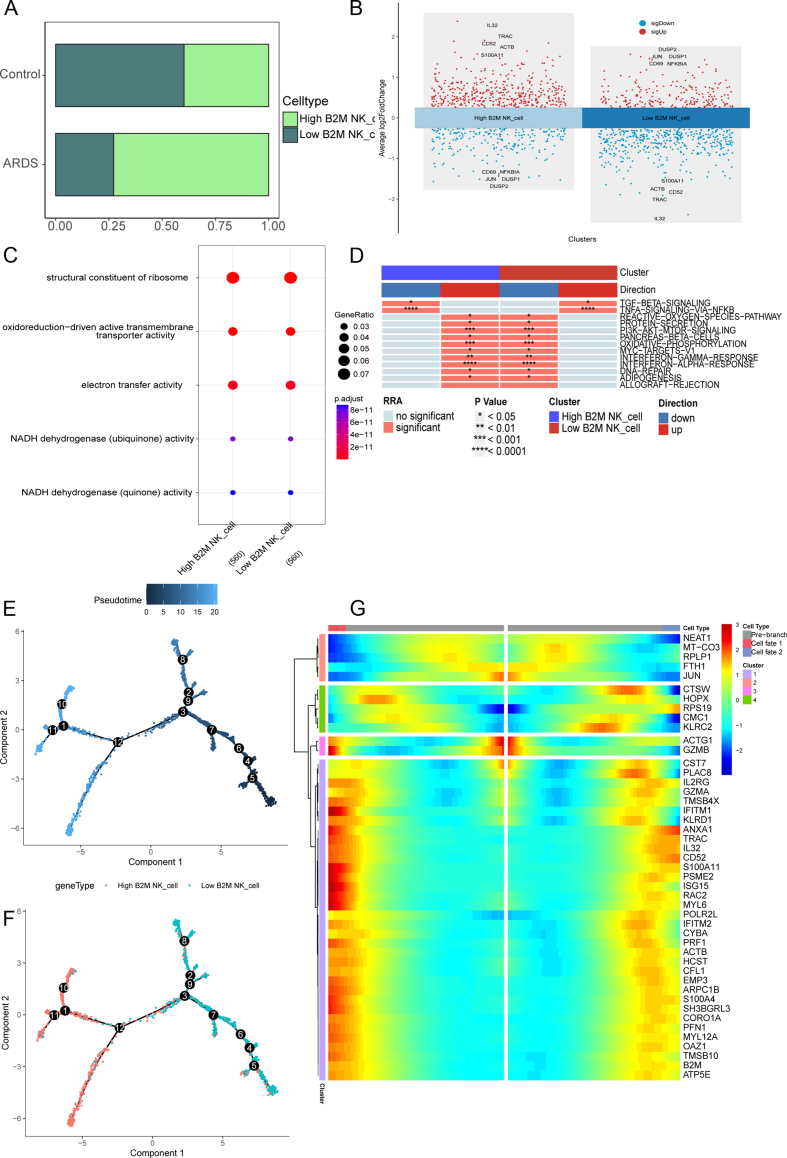
B2M function in natural killer (NK) cells. **(A)** The proportion of high B2M-expressing NK cells was significantly increased in the sepsis-induced acute respiratory distress syndrome (ARDS) group compared with the sepsis group. **(B)** Top five differential genes between high and low B2M expressing NK cells. **(C)** GO and KEGG pathways of differentially expressed genes. **(D)** irGSEA of differentially expressed genes. **(E, F)** Pseudo-time trajectory analysis indicated that B2M high-expressing NK cells were in a more advanced stage of differentiation. **(G)** Branched heat map of the relative expression of genes along the inferred pseudo-time axis.
