# Supplementary material for: Integrating single-cell RNA sequencing and bulk RNA sequencing data to predict acute respiratory distress syndrome in sepsis patients
Source: Genes Dis. 2024 Mar 19;12(1):101271. doi: 10.1016/j.gendis.2024.101271 (PMC11550751; doi:10.1016/j.gendis.2024.101271)
Supplement: Multimedia component 1 [file mmc1.docx]

**Materials and methods**

**Data source**

Bulk RNA-seq data (GSE66890) and scRNA-seq data (GSE151263) were downloaded from the GEO (https://www.ncbi.nlm.nih.gov/) database. Among the 57 sepsis patients included in the GSE66890 data, one patient's data was excluded due to a large data bias during WGCNA analysis. From the remaining 56 patients, 28 developed ARDS. Core cells were obtained by applying filtered scRNA-seq using the R package "Seurat." Cells with less than 200 genes were considered low quality and excluded from the analysis.

Principal component analysis (PCA) was performed using 15 principal components (PCs) with a P-value threshold of less than 0.05. The resulting PCs were selected for further analysis. Subsequently, the UMAP algorithm was applied to identify 15 independent cell clusters. To annotate these clusters, marker genes were identified using the "SingleR" software package, resulting in the identification of 7 distinct cell clusters. Within the NK cells, monocytes, and T cells clusters, further subclusters were classified. Marker genes were identified using the FindAllMarkers function with the parameters only.pos = FALSE, min.pct = 0.25, and thresh.use = 0.25.

**WGCNA analysis**

To identify sepsis-induced ARDS-related genes, the R package WGCNA was utilized. Initially, the hclust function of the "WGCNA" package was employed to perform hierarchical cluster analysis, enabling the identification and exclusion of abnormal samples. The appropriate soft threshold was determined by ensuring that the topology reached 0.9. Additionally, the MEDissThres was adjusted to 0.3, and a minimum of 50 genes were set for each module. Subsequently, the association between module genes and sepsis-induced ARDS was examined using Pearson's correlation coefficient.

**Construction of predictive models**

Venn diagrams were employed to display the intersection of single-cell RNA (scRNA) marker genes and sepsis-induced ARDS module genes. The genes present in this intersection were defined as sepsis-induced ARDS genes. Following that, two machine learning methods, Random Forest Graph Analysis and Support Vector Machines Recursive Feature Elimination (SVM-RFE), were utilized to conduct feature gene screening among the individual candidate genes intended for model construction. The performance of the model was assessed using the receiver operating characteristic (ROC) curve.

**Functional analysis of candidate genes and genes characteristic of sepsis-induced ARDS in scRNA sequence data**

Functional analysis plays a crucial role in unraveling the molecular mechanisms and pathways associated with sepsis-induced ARDS, thereby contributing to a better understanding of its pathogenesis. In this study, the AddModuleScore method was employed to calculate scores for 66 candidate genes across various cell and disease groups. The primary aim was to evaluate the gene set in sepsis and sepsis-induced ARDS patients, highlighting any differences, and to identify potentially significant cell clusters with distinctive scoring patterns. Expression of 6 sepsis-induced ARDS signature genes in different cell clusters and disease subgroups as shown in the violin plot. Additionally, these cell clusters were further classified into high and low expression groups based on the median B2M expression in monocyte and NK cells. The identification of differential genes was accomplished using the Wilcoxon rank-sum test. To analyze the functional enrichment of the identified differential genes, gene Ontology (GO), KEGG pathways, and ssGSEA analyses were performed. Furthermore, pseudotemporal analysis of monocyte and NK cells was conducted using the Monocle 2 algorithm. The DDR Tree algorithm was utilized to downscale each of the aforementioned cell populations. The resulting "plot_pseudotime_heatmap" visualizes the differential gene modules that covaried along pseudotime.

**Statistical analysis:**

All statistical analyses were conducted using R (version 4.2.2). For between-group comparisons, the Student t-test was applied when the data followed a normal distribution^20^, while the Mann-Whitney U-test was utilized when the normal distribution assumption was not met. Statistical significance was defined as p < 0.05.
